# Recent Advances on Porous Siliceous Materials Derived from Waste

**DOI:** 10.3390/ma16165578

**Published:** 2023-08-11

**Authors:** Daniele Montini, Claudio Cara, Massimiliano D’Arienzo, Barbara Di Credico, Silvia Mostoni, Roberto Nisticò, Luca Pala, Roberto Scotti

**Affiliations:** 1 Department of Materials Science, University of Milano-Bicocca, INSTM, Via R. Cozzi 55, 20125 Milano, Italy; d.montini1@campus.unimib.it (D.M.); massimiliano.darienzo@unimib.it (M.D.); barbara.dicredico@unimib.it (B.D.C.); silvia.mostoni@unimib.it (S.M.); 2 Fluorsid S.p.A., Strada Macchiareddu 2a, 09032 Assemini, Italy; claudio.cara@fluorsid.com (C.C.); luca.pala@fluorsid.com (L.P.)

**Keywords:** agricultural waste, inorganic materials, hexafluorosilicic acid, porous materials, silica

## Abstract

In recent years, significant efforts have been made in view of a transition from a linear to a circular economy, where the value of products, materials, resources, and waste is maintained as long as possible in the economy. The re-utilization of industrial and agricultural waste into value-added products, such as nanostructured siliceous materials, has become a challenging topic as an effective strategy in waste management and a sustainable model aimed to limit the use of landfill, conserve natural resources, and reduce the use of harmful substances. In light of these considerations, nanoporous silica has attracted attention in various applications owing to the tunable pore dimensions, high specific surface areas, tailorable structure, and facile post-functionalization. In this review, recent progress on the synthesis of siliceous materials from different types of waste is presented, analyzing the factors influencing the size and morphology of the final product, alongside different synthetic methods used to impart specific porosity. Applications in the fields of wastewater/gas treatment and catalysis are discussed, focusing on process feasibility in large-scale productions.

## 1. Introduction

According to the European Directive 2008/98/EC Article 3, the term “waste” defines “any substance or object that the holder discards, or intends, or is obliged to discard” [1]. Hence, wastes potentially represent a high loss of resources in the form of both materials and energy. In addition, the management and disposal of waste can have serious impacts on the environment [2,3]. For example, landfilling takes up land and can cause air, water, and soil pollution [4]. In 2020, the EU total waste generation from all economic activities and households was approx. 2150 million tons, corresponding to ca. 4810 kg per capita. In the same year, the European Commission adopted the Circular Economy Action Plan (CEAP), funding the transition to a circular economy, promoting sustainable consumption through targeted design and manufacture of products, and ensuring limited waste generation, while trying to preserve the resources used in the EU economy for as long as possible. In this context, companies and manufacturers are facing new challenges and regulations to reduce their environmental impact, leading to new perspectives in laboratory- and industrial-scale research with a remarkable growing attention toward novel recycling processes, aimed at converting waste from various production into useful chemicals and materials [5,6,7,8,9,10,11,12]. A bibliometric analysis on the Scopus database reveals a sharp increase in interest within the last ten years on the possibility to recover waste to convert it into nanomaterials as a way to significantly valorize it. In detail, the yearly number of publications filtered by using the terms “waste” and “nanomaterials” as keywords, passed from 12 for 2012 to 114 for 2022, with 527 total published papers in this time range. Among the most used chemicals, silica (SiO_2_) represents a fundamental material in many industrial applications due to its interesting morphological, physical, and chemical properties [13,14,15,16,17,18,19]. Several studies reported and described how it is possible to obtain SiO_2_ nanoparticles (NPs) with different aspect ratios and textural properties, involving different types of porosities and high active surface areas [20,21,22,23,24,25,26,27]. Furthermore, its chemical inertness and insulating features favor the exploitation of SiO_2_ in a plethora of technological applications [28,29,30,31,32,33]. Among these, SiO_2_ is widely applied in nanocomposite formulations (as filler) [34,35,36], industrial catalysis (as substrate) [37,38,39], wastewater remediation processes (as adsorbent) [40,41], as a building material component [42,43], and in different advanced biomedical applications (e.g., as a drug-carrier system) [44,45,46]. At the laboratory scale, the typical precursors of SiO_2_ are alkoxysilanes, such as tetraethyl orthosilicate (TEOS), or tetramethyl orthosilicate (TMSO), largely adopted in synthetic protocols following the sol-gel method [47,48,49]. At the industrial scale, precipitated SiO_2_ is synthesized by heating sodium carbonate (Na_2_CO_3_) powder with quartz sand at around 1300 °C to form sodium silicate (Na_2_SiO_3_) before reacting with sulfuric acid (H_2_SO_4_) [50]. In general, approx. 0.5 tons of Na_2_CO_3_ and 0.5 tons of H_2_SO_4_ are typically used to produce 1.0 ton of SiO_2_, with a consequent formation of byproducts, such as 0.2 tons of carbon dioxide (CO_2_), 0.7 tons of sodium sulphate (Na_2_SO_4_), and 20 tons of wastewater [50,51].

Another form of SiO_2_ that is widely used in the industry is fumed SiO_2_, which is produced by burning volatile chlorosilanes, mainly silicon tetrachloride (SiCl_4_), in a H_2_/O_2_ flame at an operational temperature of 1000 °C [38]. The reaction proceeds as follows (Reaction (1)):

2H_2_(g) + 2O_2_(g) + SiCl_4_(g) → SiO_2_ + 4HCl (1)

This process allows obtaining, at first, molten SiO_2_ nuclei, which subsequently grow into fused, spherical, non-porous sub-particles (diameter: ca. 740 nm; density: ca. 2.2 g cm^−1^) as the reaction proceeds [52].

Both high-temperature melting and the partial sintering of these sub-particles lead to their agglomeration into larger, mesoporous SiO_2_ aggregates with a high specific surface area (SSA, 50–380 m^2^ g^−1^) and a broad particle size distribution [52,53]. Finally, the synthesized SiO_2_ is isolated from the HCl vapor phase, and generally purification is firstly achieved by a mechanical method (e.g., either cyclones or filters), and subsequently completed by treating SiO_2_ with water-saturated hot air [54].

Although these methods are widely used to date, it is evident that both processes are energy-consuming routes, require harmful chemicals, (chemical and economic) unsustainable precursors, and contribute to severe environmental issues [38,50]. For these reasons, in recent years, the scientific literature has reported a large number of methodologies for the recovery of SiO_2_ from either waste or non-conventional (but greener) precursors [55,56,57,58,59,60,61], underlying the growing interest for this theme and its environmental and economical relevance. Indeed, a bibliometric analysis using the terms “industrial waste” and “silica” as keywords reveals an overall number of 454 papers published between 2012 and 2022, with a doubled number of publications uploaded yearly on the Scopus database. The recovery and/or reuse of SiO_2_ from waste deriving from productive activities and municipalities should be recognized as an important source of revenue, and this is particularly true considering the already discussed wide range of SiO_2_ applications with high added value [61,62,63].

In recent years, promising alternative SiO_2_ precursors for large-scale applications have also been represented by industrial process intermediates or secondary products, such as hexafluorosilic acid (FSA), a hazardous and corrosive by-product of the fluorine and phosphate industry [64]. However, it is mandatory to point out that the use of different waste sources as precursors might alter the final properties and characteristics of the resulting SiO_2_, strongly related to the presence of impurities, which depend on the origin and treatment of the precursors.

Hence, this study, reports on the most common synthesis procedures for obtaining SiO_2_ from waste and secondary products, highlighting the initial precursor’s composition and the pre-treatments necessary. The following discussion is organized in relation to the types of resources: firstly, it reports the isolation of SiO_2_ from agricultural and industrial waste; secondly, it provides an analysis on the synthesis and recovery procedure of SiO_2_ from FSA. The literature is selected by searching for the terms “industrial waste” and “silica” in the Scopus database in recent years (2018–present). Due to the large number of publications available, only the main relevant ones are selected, according to the authors’ sensibilities and competencies. Particular attention is dedicated to the scale-up feasibility of the presented synthetic methods in relation to the precursor nature and synthetic procedures. Finally, an insight into recent applications of waste-derived SiO_2_ is provided, with a particular focus on porous materials and their implementation in fields of scientific interest, such as wastewater treatment, pollutant adsorption, and photo-catalysis.

## 2. Silica from Industrial Production Waste or End-of-Life Products

In recent years, a significant number of studies reported on the possibility of extracting SiO_2_ from unconventional resources with the aim of reducing the environmental impact resulting from its chemical synthesis, resource wastage, and landfill use [65,66,67]. The general procedure for a correct exploitation of these alternative chemical routes requires a preliminary grinding of the starting waste to facilitate the additional steps by reducing the precursor dimensions [68], followed by further pre-treatments, such as water washing, acid washing, and incineration, to remove the highest number of contaminants [69]. In this context, this section analyzes the different types and extent of treatments, considering the nature and origin of the waste sources.

### 2.1. Agricultural Waste

In the last 50 years, agricultural production has more than tripled and the reasons of such an expansion of land dedicated to agricultural use is primarily due to the technological contribution of the green revolution that affected productivity, consequently accelerated by population growth [70]. The actual global agriculture production has been estimated to be approx. 23.7 million tons of food per day [71]. The rapid growth of global production has significantly affected the environment causing considerable large stress, with negative impacts on soil, air, and water resources, and consequently on population health [72,73,74].

The management of agro-industrial waste is one of the tools to mitigate the impact of agriculture production and, in this context, an analysis of the scientific literature highlighted the possibility of recovering SiO_2_ from them. Among the different precursors obtained from agricultural sources, the majority of the studies reported the use of sugar cane bagasse [75], bamboo leaves [76], wheat straw [77,78], corncob [79,80], and rice husk [30,81,82,83,84]. In particular, rice husk is the most promising precursor due to its high abundance (the worldwide annual rice production is approx. 120 million tons [85]). Rice husk composition depends on variety, origin, climate, and geographical location [86]; however, with respect to other biomasses, its initial SiO_2_ content can reach values >90 wt.% of the total inorganic residue (Table 1) [75,76,77,78,79,80,82,84,87].

**Figure 1:**
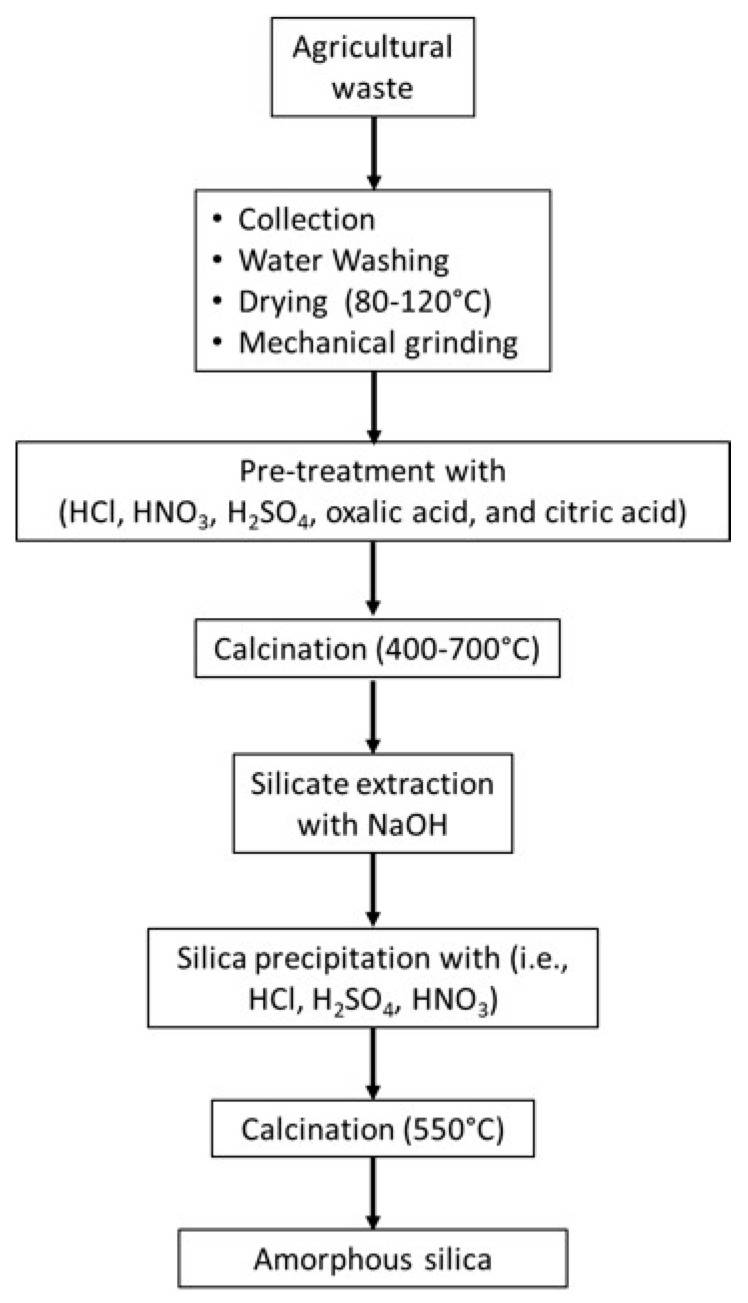
Schematic representation of a typical process for the production of SiO_2_ from agricultural waste.

Most of the impurities found in these agricultural wastes are represented by residual organic matter (such as hemicellulose, cellulose, and lignin, covering 70–85 wt.%, depending on the type of biomass), whereas the remaining part consists of transition and alkali oxides, such as MgO, CaO, K_2_O, Na_2_O, and other minerals (i.e., aluminum oxide) [88]. After collecting the agricultural waste, the starting biomasses are preliminary washed and dried [89]. An initial preliminary purification step is usually performed, washing with either deionized water, coupled with either acid leaching or thermal treatment at high temperatures (Figure 1).

Acid leaching is a treatment useful for removing metallic impurities [90]. According to the literature, there are a variety of methods for this purpose, typically based on the use of HCl [84], HNO_3_ [91], H_2_SO_4_ [92,93], oxalic acid [94], and citric acid [95], all at various concentrations [94]. Thermal treatments, instead, involve pyrolysis, which consists of incineration processes performed at high temperatures (400–1000 °C), thus aiming at removing organic impurities, leaving SiO_2_ and the mineral components [96,97]. The drawback of pyrolysis is the release of volatile byproducts that may contribute to environmental pollution [98]. This cleaning step involving a combination of chemical and thermal treatments producing agricultural waste ashes that generally contain ca. 85–95 wt.% of SiO_2_ [99]. However, the final material purity can be further extended up to ca. 95–98% with additional alkaline extractions and washing steps.

An example of SiO_2_ production from agricultural waste is the recent work of Akhayere et al. [100], which reported the synthesis of SiO_2_ NPs from barley husk, describing the typical preparation and pre-treatment of samples. In this work, barley husk, cut into small pieces and rinsed with water, was dried at 100 °C for 24 h and, subsequently, ball milled and refluxed in a 2M HCl solution for 6 h. Both pre-treatment and purification steps involved filtration, washing with deionized water, and final heating in air at temperatures ranging from 400 to 700 °C.

The purity of the resulting nanoscopic SiO_2_ was 93–94% (Table 2), depending on the thermal treatment conditions, from 400 to 700 °C, respectively [100]. Furthermore, the mechanical properties of SiO_2_ were dependent on both thermal treatment conditions and SiO_2_ purity. The authors underlined that a higher purity could be reached by performing thermal treatments at higher temperatures; however the calcination of the barley husk beyond 700 °C may lead to the conversion of amorphous SiO_2_ into its crystalline polymorphs (e.g., quartz, cristobalite, and trydimite) causing particle agglomeration and reducing the active surface area of the barley husk ash’s SiO_2_. Thus, the temperature-sensitive nature of ash-derived SiO_2_ requires suitable combustion apparatus and pyrolysis technology, with controlled combustion temperatures and times, for a large-scale industrial production. However, even under controlled calcination conditions, the internal heat of the husk heap was difficult to release, with the risk of rapidly exceeding the crystallization point of SiO_2_. Therefore, reducing the crystallization sensitivity of ash-derived SiO_2_ through the control of the calcination temperature is another critical issue for achieving a large-scale production of SiO_2_ from this type of biomass.

**Figure 2:**
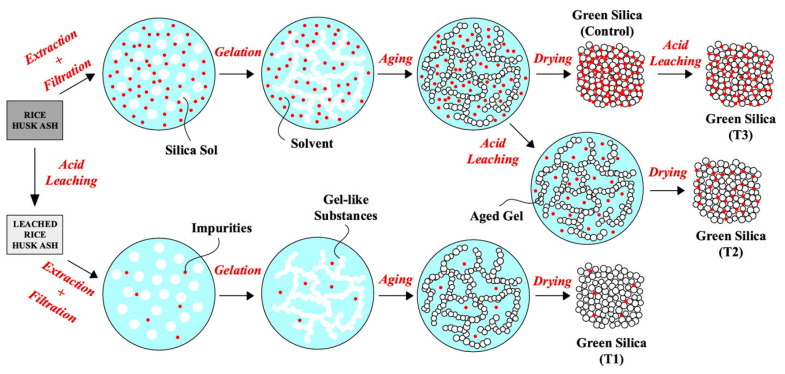
Schematic representation showing a different (alternative) purification route for rice husk ash for the growth and isolation of SiO_2_ NPs. Reprinted with permission from [83].

Additionally, the acid leaching step had an effect on both the purity and superficial properties of rice husk SiO_2_. Steven at al. [84] investigated the effect of different acid leaching sequences to find the optimal conditions to improve SiO_2_ quality for industrial purposes (Figure 2).

The sample treated with 1M HCl at a temperature of 100 °C for 1 h showed the highest purity and surface area, reaching 96.4% and ca. 400 m^2^ g^−1^, respectively. The advantage of using HCl despite the other acid resides was its easy handling and availability. However, the authors also reported that the use of acid as a leaching agent caused the corrosion of pipes and instruments, thus increasing the operational difficulties and production costs, making the large-scale application of these processes tricky and inconvenient. To face these challenges, Xu et al. [87] presented a novel and more environmentally friendly process based on a water leaching pre-treatment process to extract highly reactive SiO_2_ from rice husks. The authors reported that the boiling-water leaching pre-treatment of rice husks significantly removed the metallic impurities and reduced the crystallization sensitivity of rice husk SiO_2_ to a calcination temperature, yielding amorphous SiO_2_ with a purity of ca. 94%, comparable to the samples pre-treated with HCl, H_2_SO_4_, and HNO_3_. Furthermore, the resulting SiO_2_ remained amorphous even after thermal treatment at 900 °C for 7 h, even if with smaller surface areas (i.e., 130–138 m^2^ g^−1^) compared to acid treatments (i.e., 200–400 m^2^ g^−1^).

An alternative approach to produce SiO_2_ from agricultural waste is the direct combustion of biomass. In this case, a sufficient air flow is required for exerting complete combustion, avoiding the presence of unburned carbon in the resulting ashes [101]. The combustion temperature and heating rate are crucial parameters. In particular, by increasing the heating rate, an increment of the SSA and pore volume of the remaining ashes occurred as far as a reduction in SiO_2_ purity and brightness. Interestingly, a multi-step decomposition of rice husk and rice straw was observed during the laboratory studies to obtain high-purity SiO_2_ with a low-carbon content and high SSA (>200 m^2^ g^−1^) [102,103]. Specifically, the first stage corresponded to drying (50–200 °C) for the removal of physically bonded water, the second stage involved the burnout of volatile organic components with (200–340 °C), and the final stage was due to the degradation of carbonaceous phase cellulose and hemicellulose (340–500 °C). In the literature, it is possible to find some attempts to scale-up this technique at the level of a pilot plant [104,105].

This strategy guarantees the amorphous structure of SiO_2_ since the operating temperature of the plant (600–800 °C) can be kept below the SiO_2_ crystallization temperature [103,106,107]. However, even if this method can reach a productivity of 100 kg h^−1^ of raw rice husks, preserving the resulting SiO_2_ from self-aggregation phenomena and maintaining high SSA (compared to the alkaline extraction method [108]), the further upscaling of this combustion process at the industrial level still presents some technological limitations, such as the need for time-consuming procedural sequences [109,110,111].

### 2.2. Industrial Waste

Industrial waste is defined as the waste generated by industrial activities, including all materials rendered unusable during a manufacturing process, such as the ones occurring in factories, industries, mills, and mining operations. In general, this type of waste can be divided into hazardous and non-hazardous [112]. Hazardous wastes are residues that may harm public health and/or the environment, e.g., combustible, corrosive, active, and toxic materials [113], whose production in the EU-27 reached ca. 101.4 million tons in 2018 [114]. In contrast, non-hazardous wastes do not pose a risk to public health and/or the environment, e.g., cardboard, plastic, metals, glass, rock, and organic waste [115].

Industrial waste SiO_2_ sources that have attracted the attention of the scientific community in recent years are the bottom and fly ash of different productions [116,117,118,119], blast furnace slag [120,121], and photonics industry waste [122,123]; however, one of the most environmentally impacting industries that produce siliceous waste and must be taken into consideration is certainly the mining industry [124,125,126,127,128,129,130]. In fact, in the extraction of minerals and ores, SiO_2_ is often a major component of the waste residue [131]. For example, the SiO_2_ content of post-flotation waste deriving from copper ores is between 27% and 59%, whereas SiO_2_ is the main component in the ore tailings from iron-extraction SiO_2,_ also reaching values close to 90 wt.% (the estimated amount of iron ore discarded every year is ca. 130 million tons [132]). The general composition of different mining wastes is reported in Table 3 [133,134,135,136,137,138].

**Figure 3:**
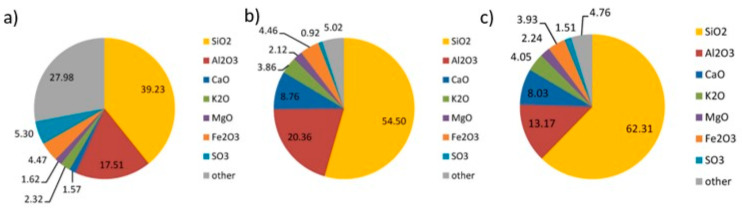
Mineralogical compositions of coal post-flotation waste (**a**), coal ash (**b**) and of cuttings from the oils and gas sector (**c**). Numerical values indicate the percentage. Reprinted with permission from [139].

A constant concentration of SiO_2_ is also found in other mining activities, such as coal and mineralogical compounds used in oil and gas production, with average concentrations of 55% and 62%, respectively (Figure 3) [139].

Even if considerable efforts are made to recycle mining waste for a variety of technological applications [132,140,141,142,143], there are still a few examples of its application in the extraction of nanosized SiO_2_, and most of the studies concentrate their efforts on producing mesoporous SiO_2_ with the aim to provide the recovered material with a functionality of high technological interest. In this case, the main concern is the contamination by other metal oxides, and the most common technique used for pre-treatment is acid leaching. In 2014, for the synthesis of mesoporous SiO_2_ from iron ore residues, Yang et al. [134] applied an acid leaching pre-treatment with HCl (30 wt.%) at ca. 100 °C for 1.5 h to extract iron species; then, FeCl_3_ was removed by filtration. The acid-insoluble material had a SiO_2_ content of almost 80 wt.% that was additionally purified by converting it in soluble silicate under alkaline conditions and then by reprecipitating it under acid conditions. A mesoporous structure was achieved by using cetyltrimetilammonium bromide (CTAB) as a templating agent. The details of the porosity types and methods are discussed in paragraph 3 (vide infra).

As an example, Fu et al. [133] reported on the production of mesoporous MCM-41 SiO_2_ using sodium silicate derived from copper ore with a high Fe_2_O_3_ content. The method allowed extracting SiO_2_ with a minor content of iron impurity and consisted of mixing the copper ore with NaOH and NaNO_3_ with a weight ratio of 5:5:3. After the calcination step, the powder was mixed with deionized water (1:4 *w*/*w* ratio) and the leaching of the sodium silicate was performed by stirring at 100 °C for 6 h. Finally, a mesoporous structure was obtained by introducing CTAB to the extracted sodium silicate solution and by performing calcination at 500 °C for 6 h. The material still contained some impurities, such as Al_2_O_3_ (1.230%), Na_2_O (0.670%), Fe_2_O_3_ (0.071%), and Cu (0.007%). To achieve a higher degree of purity for this type of waste, an alternative pre-treatment method involving high-gradient superconducting magnetic separation able to remove the iron content was performed at the laboratory scale [144]. Yang et al. [144] demonstrated an increase in SiO_2_ content from 68.7% to 92.6 wt.% by applying this technique to iron ore tailings. Additionally, a further improvement of the SiO_2_ content (reaching 99.9 wt.%) was obtained by adding an acid leaching step with a mixture of HNO_3_, HCl, and H_2_SO_4_ (a molarity ratio of 1:4:1, solid–liquid weight ratio of 1:4, and leaching temperature of 80 °C) [145]. However, the proposed process showed some important disadvantages, such as low efficiency, insufficient utilization of raw materials, and pollution as the main issues.

### 2.3. Work-Up Procedures for Agricultural and Industrial Waste

**Figure 4:**
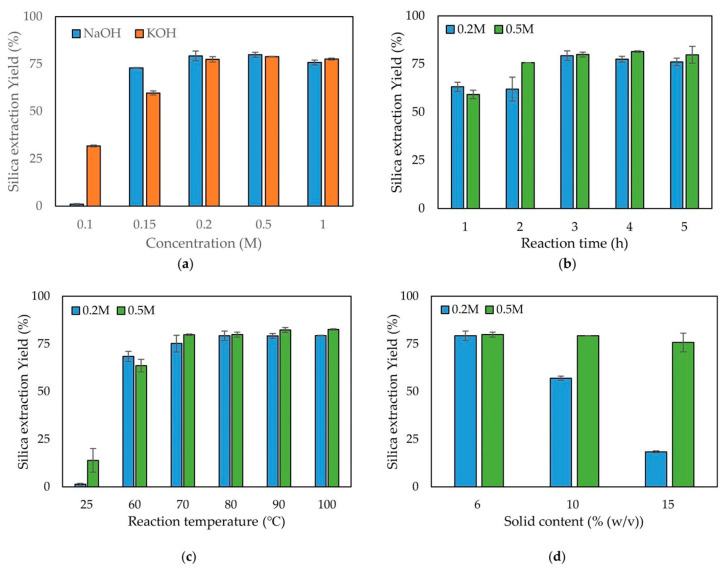
Comparison of the different SiO_2_ extraction yields depending on (**a**) the type of alkaline solution (NaOH vs. KOH); (**b**) the alkaline leaching reaction time (from 1 h to 5 h); (**c**) the temperature (from 25 °C to 100 °C); and (**d**) the solid content (from 6% to 15%). Reprinted with permission from [82].

The majority of the cases reported in the literature involving the recovery of high-purity SiO_2_ from different waste sources requires a chemical extraction generally using an alkaline agent (in this case, followed by acid neutralization). In a typical alkaline extraction process, a two-step approach is followed, namely, (i) the alkaline dissolution of the waste source resulting from the cleaning process in a base (e.g., NaOH, KOH) and (ii) SiO_2_ precipitation by using an acid until reaching neutralization [81,146,147]. Park et al. [82] evaluated the performances of two different alkaline extraction methods for SiO_2_ recovery from rice husks by using either NaOH or KOH solutions at different concentrations. At an alkaline concentration of 0.1M, the SiO_2_ extraction yields were 10% and 30% for NaOH and KOH, respectively, thus evidencing the higher extraction yield of KOH at a low concentration. Furthermore, by increasing the concentrations of the alkaline solutions, the extraction yields increased up to approx. 75–80% (Figure 4) and, above this point, became independent from the alkaline concentration (saturation level).

In the case of NaOH extraction, the maximum extraction yield (79%) was achieved at a base concentration of 0.2M, whereas with KOH this was achieved at 0.5M (77%). This highlights that NaOH is more convenient to maximize the extraction yield at a lower concentration of the alkaline media.

In another study, Haq et al. [148] reported that the optimal concentration of NaOH for maximizing the SiO_2_ extraction yields from rice husk ash was in the 0.8–1M range and it was subjected to the regional variation of rice husk, which affected the SiO_2_ content. The authors pointed out that fundamental parameters for an optimal extraction yield, both at laboratory and process industrialization scales, were (i) the duration of the extraction, with a maximum yield after 90 min and no further improvement at longer times, and (ii) an NaOH/rice husk ash mass ratio with optimal values in the 0.01–0.06 range.

Many literature studies pointed out that, in addition to varying times, the differences in extraction yields could be achieved by varying temperatures, according to the origin, nature, and impurities of the waste. The temperature for silicate extraction ranges from room temperature (RT) to 120 °C, generally under reflux conditions; however, it is difficult to find a general guideline. In fact, in the case of the incineration of bottom ash, Alam et al. [149] found that with an ash:NaOH mass ratio of 1:0.8, a low-temperature (i.e., 20 °C) condition for 24 h led to the incomplete dissolution of SiO_2_, whereas the optimal extraction was achieved at a higher temperature and longer time (i.e., 75 °C and 48 h, respectively). Zhao et al. [150], instead, successfully treated bauxite reaction residue from the polyaluminum chloride (PAC) coagulant industry with 3M NaOH and a liquid:solid ratio of 1:5, achieving an optimal extraction yield of ca. 81.5% at 75 °C, analogously to Alam et al. [149], but in a shorter time (ca. 4 h). The same study evidenced that comparable yields could be obtained by reducing the time (ca. 2 h) and increasing the extraction temperature to 90 °C. This result is very appealing for a large-scale application, as it discloses the possibility of properly manipulating experimental parameters to achieve the best trade-off between the product yield and resource consumption.

Finally, in the case of the alkaline extraction, there were some additional technological issues that should have been considered, such as the solution’s corrosiveness, high-cost implication, complexity of SiO_2_ recovery, environmental issues related to the use of strong bases, and difficulty in eliminating the byproducts (i.e., Na_2_SO_4_, NaCl) entrapped in the SiO_2_ nanopores [69,151].

As previously anticipated, the alkaline extraction was followed by SiO_2_ precipitation due to a pH drop obtained by slowly pouring an acid into the alkaline solution after silicate formation, maintaining a constant temperature. Among the different types of acid, HCl was the most used; however, other acids (such as H_2_SO_4_, H_3_PO_4_, HNO_3_, CH_3_COOH, and citric acid) were applied. Then, washing and neutralization with deionized water is usually performed, followed by a subsequent drying step. Eventually, in the case of microporous and mesoporous nanomaterials, a further calcination step is performed at 550 °C for 6–8 h to remove organic templates [152,153].

In summary, an examination of the recent literature reveals that there is no consistent homogeneity in the conditions, leading to variations in the optimal parameters and synthetic approaches, in relation to waste origin, nature, and desired SiO_2_ final characteristics. Regarding the economic feasibility of these processes, different factors must be evaluated, such as the abundance of waste, extraction costs, type, and purification step. Particularly, this last step of SiO_2_ production is where the process differs the most, with respect to the industrial production of SiO_2_ from precipitation, and where the cost competitiveness could reside. For this reason, an economical and cost–benefits analysis is fundamental in consideration of the type of waste, origin, and region of application. Most published patents on the topic concern the synthesis of biogenic amorphous SiO_2_, showing its high applicative potential as a filler for rubber and concrete industries, which is discussed in paragraph 3 (vide infra).

However, at present, these processes still have some limitations to their extensive applications. In fact, the extraction and processing of SiO_2_ from rice husk can be more expensive compared to traditional sources of SiO_2_, such as quartz and sand, thus demanding investments and innovations for making the process viable for large-scale applications. The availability of waste in large quantities and proximity to processing facilities are also important factors in determining the feasibility of large-scale SiO_2_ production. In some regions, the abundance of waste may not be sufficient to justify an industrial scale-up. Another aspect to take into consideration is that ensuring the consistent quality and purity of SiO_2_ extracted from agricultural/industrial waste, as addressed in the previous paragraphs, can be challenging, and, in addition, there is a general lack of legislation regulating the use of waste as a raw material and re-introducing waste-derived materials into the market. In relation to this last point, a very promising approach is represented by the possibility to obtain SiO_2_ directly from Si-rich byproducts from different industrial manufactures in an integrated process alongside the principal production line.

### 2.4. Silica Recovery from Hexafluorosilic Acid

FSA is a hazardous byproduct from the phosphate and fluorine-derivatives industries whose worldwide production is estimated at approx. 2 million tons per year [64]. The main concern regarding FSA production is its disposal, which is mostly performed by neutralization directly into the sea [154]. In fact, FSA storage is quite expensive and potentially dangerous as FSA violently reacts with bases and water-producing HF, and it is extremely corrosive against metals and glassware [155]. FSA spillage in a water body affects the local ecosystem reducing the pH level and increasing the fluorine concentration. Due to its limited market (i.e., FSA is typically used in some niche applications, such as either water fluorination or the production of AlF_3_) and the increasing legislation to regulate its disposal, both industrial and academic research are focusing their attention on the possible reuse of FSA, in particular, the synthesis of various Si-based materials [156,157,158].

In the phosphate and fertilizer industries, FSA is produced during the leaching of phosphate ore by sulfuric acid, which forms a gas stream of SiF_4_ and HF purified via adsorption in a water scrubber, producing a solution of FSA maintained at a fixed concentration by the addition of water (typically 24 wt.%) [159]. The reaction mechanism is as follows:

Ca_5_(PO_4_)_3_F + 5H_2_SO_4_ + nH_2_O → 3H_3_PO_4_ + 5CaSO_4_·nH_2_O + HF (2)

4HF + SiO_2_ → SiF_4_ + 2H_2_O (3)

3SiF_4_ + 2H_2_O → 2H_2_SiF_6_ + SiO_2_ (4)

Conversely, concerning the fluorine-derivatives industry, and, in particular, HF production, FSA is obtained from a direct reaction between HF and SiO_2_ impurities (0.5–1.5 wt.%) contained in feedstock, namely, acid grade fluorspar (CaF_2_).

CaF_2_ + H_2_SO_4_ → CaSO_4_ + 2HF (5)

4HF + SiO_2_ → SiF_4_ + 2H_2_O (6)

3SiF_4_ + 2H_2_O → 2H_2_SiF_6_ + SiO_2_ (7)

SiF_4_ + 2HF → H_2_SiF_6_ (8)

Reaction (6) presents the formation of SiF_4_, which in turn generates FSA by a reaction with either water in the scrubber (Reaction (7)) or HF (Reaction (8)).

At the laboratory scale, the recovery of FSA usually occurs through an acid–base reaction, typically using NH_3_, Na_2_CO_3_, or NaOH. In Reaction (9), ammonium hexafluoro silicate (NH_4_)_2_SiF_6_ formed as an intermediate after an ammonization reaction (Reaction (10)), leading to ammonium fluoride (NH_4_F) and SiO_2_ precipitation.

H_2_SiF_6_ + 2NH_4_OH → (NH_4_)_2_SiF_6_ + 2H_2_O (9)

(NH_4_)_2_SiF_6_ + 4NH_4_OH → SiO_2_ + 6NH_4_F + 2H_2_O (10)

The type of feedstock and reactants used for the FSA production led to the formation of different secondary products that could persist as traces in FSA-derived SiO_2_. Ayarza et al. [160] reported a new method for identifying impurities present in FSA implementing capillary zone electrophoresis, a method that could be applied at an industrial scale due to its simplicity and accuracy. In this study, the authors quantified as principal impurities the presence of chlorides and sulfates (both at a concentration of approx. 1220 mg L^−1^). Furthermore, this method also allowed to qualitatively determine the presence of other inorganic anions, such as bromides and nitrates.

To the best of our knowledge, the scientific literature does not reported the studies discussing purification steps performed at the beginning of the process. In the following paragraph, a critical discussion focusing on the possible synthesis variations, fluorine leftover, and different post-synthesis treatments are presented.

In 2010, Sarawade et al. [156] studied FSA conversion into SiO_2_ through a reaction with 0.3M Na_2_CO_3_. To obtain a slurry at different pH levels (in the 1–10 range), the reaction temperature was set at 100 °C. The slurry pH was modified before filtering SiO_2_, which was successively redispersed and aged in water at 80 °C. The resulting amorphous SiO_2_ was finally recovered by filtration, washed with water, and dried at 150 °C. The proposed mechanism of SiO_2_ formation involves the reaction of FSA with Na_2_CO_3_ in an aqueous solution to form unstable monomeric silicic acid.

H_2_SiF_6_ + Na_2_CO_3_ → Na_2_SiF_6_ + CO_2_ + H_2_O (11)

SiF_6_^2−^(aq) + 4H_2_O(l) → Si(OH)_4_(aq) + 4 H^+^(aq) + 6F^−^(aq) (12)

In this reaction mechanism, monomeric silicic acid undergoes condensation and polymerization reactions to form siloxane bonds, and, consequently, a SiO_2_ network.

≡Si(OH) + (HO)Si≡ → ≡SiOSi≡(s) + H_2_O(l) (13)

The secondary product, sodium fluoride (NaF), is obtained from the evaporation of the remaining solution (Reaction (14)).

Na^+^(aq) + F^−^(aq) → NaF(s) (14)

The obtained solid was purified by washing it with water and then, finally, spray-dried. Nguyen et al. [157] reported a process based on the reaction between FSA and a 20 wt.% ammonia solution at RT for 12 h under mechanical stirring to obtain a slurry with suspended SiO_2_. The SiO_2_ formation mechanism reported by Vacca et al. [64] consisted of a typical acid–base reaction where the intermediate ammonium hexafluoro silicate (NH_4_)_2_SiF_6_ almost immediately reacted, precipitating as SiO_2_, leaving in the aqueous phase ammonium fluoride (NH_4_F) as a secondary product. Yu et al. [161], instead, studied the synthesis and purification of SiO_2_ obtained from FSA recovered from a fertilizer plant located in South Korea, with an extremely heterogeneous elemental composition, with Si being 4.83%. In this study, a two-step ammoniation treatment was performed first to neutralize FSA and then to recover high-grade amorphous SiO_2_. This process followed the same scheme observed in Vacca et al. [64]. In this case, the rapid ammoniation favored the formation of (NH_4_)_2_SiF_6_ limiting its conversion into SiO_2_.

(15) $$H_{2}SiF_{6}+2NH_{4}OH\to (NH_{4}{)}_{2}SiF_{6}+2H_{2}O$$

The experimental conditions were optimized allowing to recover amorphous SiO_2_ with 99.5% purity by performing a first ammoniation step with an NH_3_:FSA molar ratio equal to 3.0 and a second step equal to 4.2. In particular, the authors observed a drastic lowering of Ca, Al, and Mg impurities with this two-step procedure, whereas the presence of Fe, K, and Na impurities seemed unaffected.

In general, since the amorphous SiO_2_ obtained from the FSA conversion route presented high-grade purity, at relatively low process costs, FSA represented a very promising and valuable alternative substrate with respect to conventional SiO_2_ precursors, even for large-scale applications. The main limitation of extending the development of FSA-related chemical routes was the strong dependence of the technology being localized in only nearby plants where the precursor was generated.

## 3. Application of Waste-Derived Silica

Nanoscale SiO_2_ with different morphological and surface properties can be obtained from agriculture and industrial waste through the methods described in the previous paragraph, finding important applications in areas, such as building materials and rubber manufacturing [162,163,164,165,166,167].

Furthermore, in recent years, several studies have focused on the recovery of waste siliceous materials, imparting during the synthesis a controlled porosity, to enhance the final material value-added and applicability in high technological applications. In the following paragraphs, the descriptions of the main consolidated applications and possible future ones are reported.

### 3.1. Industrial Applications of Waste-Derived Silica

An important field of application of SiO_2_ is the cement industry [168], where SiO_2_ fume is added to Portland cement-containing concrete to improve its properties, in particular, its compressive strength, bond strength, and abrasion resistance.

In this field of application, in general, there are no strict requirements in terms of morphology and composition, which may favor the real industrial implementation of waste-derived SiO_2_.

However, the cement industry is one of the most impacting manufacturers in the world as it is estimated that, in 2019, its related overall CO_2_ emissions represented approximately 10% of global energy-related CO_2_ emissions. For this reason, replacing cement paste with waste-derived SiO_2_ in principle could help to lower the cement industry’s environmental impact. For this purpose, Luo et al. [169] recently studied the use in cement formulations of fumed SiO_2_ recovered from the smelting of ferrosilicon alloys at two different purity grades (86.3% and 96.0%). The results showed that the addition of fumed SiO_2_ significantly improved the mechanical properties of concrete, increasing its cubic compressive strength and splitting-tensile strength by 26.7% and 40.7% for low-grade-purity SiO_2_ and by 44.7% and 57.4% for purified samples, respectively. Nevertheless, the authors reported a limited cost increase per cubic meter of concrete by 1.9% for low-grade-purity SiO_2_ and by 5.3% for high-grade-purity SiO_2_; however, the competitive prices were maintained when compared with the cement in the same compressive strength class. Furthermore, the partial replacement of cement paste with waste-derived SiO_2_ can significantly reduce its ecological impact, saving 11.5 kg of cement per cubic meter of concrete.

Regarding the utilization of waste-derived SiO_2_ as fillers in rubber composites for inducing rubber reinforcement, this is one of the most economically relevant application and it represents a good opportunity for advanced waste valorization. To demonstrate the feasibility and effectiveness of the use of waste-derived SiO_2_ in rubber, Lolange et al. [170] studied the reinforcing properties of rice husk SiO_2_ compared to commercial samples prepared with conventional methods. As a first step, the rice husk ash was digested in an NaOH solution at 145–150 °C in a high-pressure reactor (3–4 bars). The obtained sodium silicate solution had a SiO_2_ percentage equal to ca. 23 wt.%. SiO_2_ precipitation was then performed with a sulfuric acid solution in the presence of an anionic size-controlling agent. SiO_2_ NPs obtained from rice husk are amorphous with a 20 nm diameter and a BET SSA of ca. 195 m^2^ g^−1^, characteristics similar to commercial samples commonly used as rubber fillers. Finally, the fillers tested in a typical nanocomposite formulation with styrene butadiene rubber (SBR) and butadiene rubber (BR) showed a significant improvement of tensile strength and elongation at break for the materials reinforced with rice husk SiO_2_ compared to the reference samples, being stronger and more resilient than the references, with comparable hardness and tear strength. Despite these excellent results, it must be considered that the use of an anionic surfactant for size control may pose problems in relation to actual extended industrial applications. However, as already observed, there are several patents on this topic [163,164,165,166,167,168], concerning both the synthesis and compounding of biogenic SiO_2_, and it is known that important industrial tire companies, such as Continental, Goodyear, and Pirelli, are trying to industrialize waste-derived SiO_2_, aiming to double their production rates in the coming years [171,172,173].

Finally, SiO_2_ can be employed in other applications, such as a paint filler, in refractory manufacturing, ceramics, and insulation, where recovered SiO_2_ is used without strict requirements in terms of morphology and composition. However, the quantities required for these manufactures are much lower than the aforementioned applications. As a result, there is a limited number of published papers and patents focusing on these particular topics.

### 3.2. Porous Waste-Derived Silica

**Figure 5:**
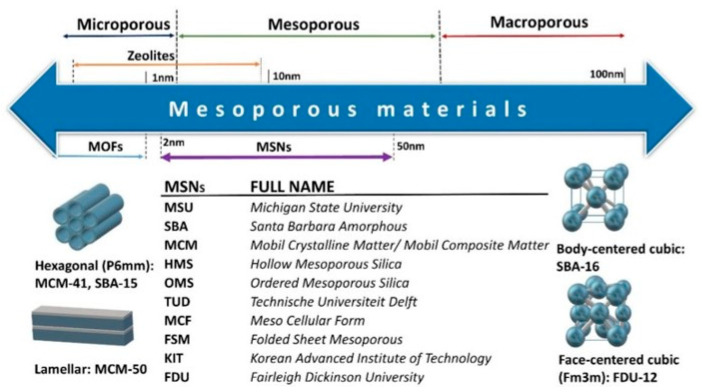
Schematic depiction of various mesoporous SiO_2_ NPs, reporting the most relevant acronyms, and the relative porous structures. Reprinted with permission from [175].

According to the International Union of Pure and Applied Chemistry (IUPAC), microporous materials have a pore size smaller than the 2 nm range and mesoporous materials in the 2–50 nm range; macroporous materials have a pore size larger than 50 nm (Figure 5) [174,175].

**Figure 6:**
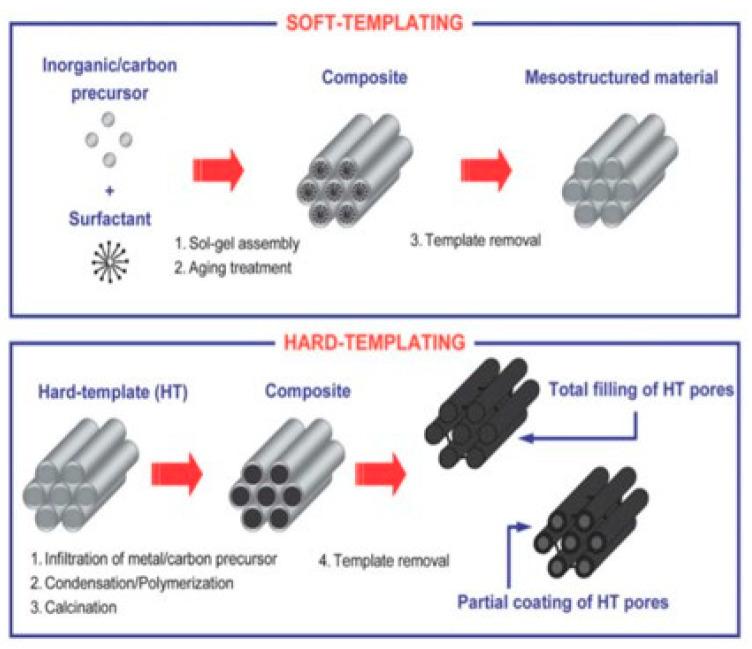
Comparison between soft-templating and hard-templating routes for obtaining ordered mesoporous materials. Reprinted with permission from [185].

Porous materials find applications in a plethora of different key fields, such as the sorption and storage of chemicals, ion exchange, catalysis, filters, biomedicine and drug-delivery systems, and thermal and acoustic insulation [174,175,176,177,178,179,180]. They present large SSAs, with many active sites available for the functionalization with molecules [181,182,183,184]. To achieve a controlled porosity, the synthesis requires the use of templating techniques (i.e., soft- and hard-templating approaches) able to drive the formation of the SiO_2_ network (Figure 6). The soft-templating approach commonly uses flexible nanostructures as surfactants, organic molecules, and block copolymers, interacting with precursors by weak non-covalent bonds, such as electrostatic or van der Waals interactions and hydrogen bonding. On the contrary, the hard templating, also known also as “nanocasting”, involves solid materials, such as polymers, metal oxides, and carbon NPs, as templating agents. Herein, the morphology of the final material was directly determined by rigid templates, removed by carbonization or acid treatments, assuring uniformity in the porous structure [185,186,187,188].

To the best of the authors’ knowledge, highly porous siliceous materials are mostly obtained by means of soft-templating routes. The most commonly used templates are amphiphilic molecules, such as surfactants (e.g., CTAB, polyethylene glycol (PEG)) [189,190,191] and/or block copolymers (e.g., Pluronic P123, F127, and poly(styrene)-block-poly(ethylene oxide) (PS-*b*-PEO)) [192,193,194,195,196,197]. During the synthesis with conventional SiO_2_ precursors, the organic templates are incorporated in the alcoholic aqueous medium under acid/basic conditions [67,75,83,198]. Some studies reported the mechanism of formation of SiO_2_ nanospheres and nanorods by modifying both the reactant’s concentration and template’s ratios [199,200,201]. By varying these parameters and, in particular, the template’s intrinsic properties (i.e., size, length, charge, etc.), it was also possible to regulate the micellar shape/dimensions and, consequently, the final morphology of the porous network [152,192,202]. In both soft- and hard-templating processes, the organic templates were removed by either chemical treatment (e.g., acid/base washing, solvent extraction, and dialysis) or thermal calcination under an air atmosphere in a furnace at 500–600 °C [75,203,204].

Mesoporous SiO_2_ NPs obtained following this approach have been extensively studied since the discovery of MCM-41 systems in 1992 by the Mobil Oil Company. Table 4 reports the different types of mesoporous SiO_2_ systems and their characteristics [205].

The scientific interest for the possibility of obtaining microporous and mesoporous SiO_2_ from waste or secondary sources has significantly increased, as testified by the numerous fields of application and the incrementing added value of the recovered products [206,207,208,209,210,211]. In the following section, some recent works were critically summarized, with particular focus on the synthesis conditions and treatments.

**Figure 7:**
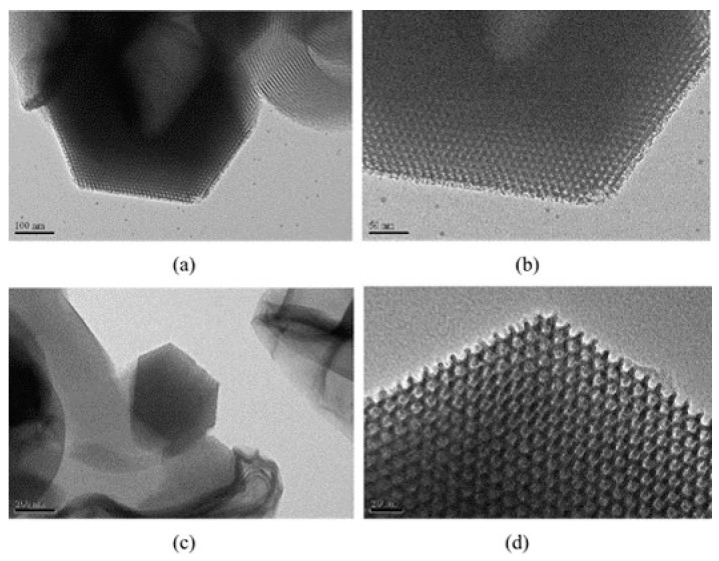
TEM images at different magnifications of: (**a**,**b**) rice husk-SBA-15 SiO_2_; (**c**,**d**) rice husk-GO/SBA-15 composite. Reprinted with permission from [212].

One of most interesting and inspected applications of these materials regards the adsorption of pollutants for water and gas treatments. Liou et al. [212] synthetized a graphene oxide(GO)/SBA-15 composite from rice husk ash demonstrating an enhanced adsorption capacity against Rhodamine-B dye due to the high number of oxygen-containing functional groups present in the material. Collected rice husks dried in an oven, and soaked in a 3M HCl solution at 100 °C for 1 h to eliminate trace metallic compounds, were washed with water and heated in an air oven at 100 °C for 24 h. Then, carbonization was conducted in a tubular furnace at 700 °C for 1 h under a highly purified N_2_ atmosphere to obtain C/SiO_2_ solids, which were successively dipped into a 1.5M NaOH solution at 100 °C for 1 h under stirring to obtain a sodium silicate solution. The hybrid material was prepared through hydrothermal treatment by adding this silicate solution to an acidic mixture of a surfactant (P123), GO, and 2M HCl under stirring for 24 h at 35 °C and treating this solution at 100 °C in an autoclave for 24 h. After the hydrothermal treatment, the powder was dried and heated at 550 °C for 6 h in a tubular furnace along with an N_2_ gas injection. The GO/SBA-15 composite showed a typical SBA-15 SiO_2_ regular porous structure, with a SSA of 625 m^2^ g^−1^ (Figure 7) [212].

The highest adsorption capacity was obtained for rice husk-GO/SBA-15, namely, 151.28 mg g^−1^ at 80 °C, a value much higher than other previously studied GO hybrid materials [213,214,215].

**Figure 8:**
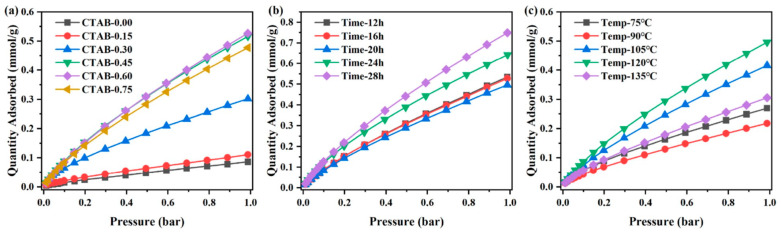
CO_2_ adsorption isotherms of different porous SiO_2_ at 298 K under the pressure range of 0.01–1 bar: (**a**) CTAB additions; (**b**) hydrothermal time; (**c**) hydrothermal temperature. Reprinted with permission from [217].

**Figure 9:**
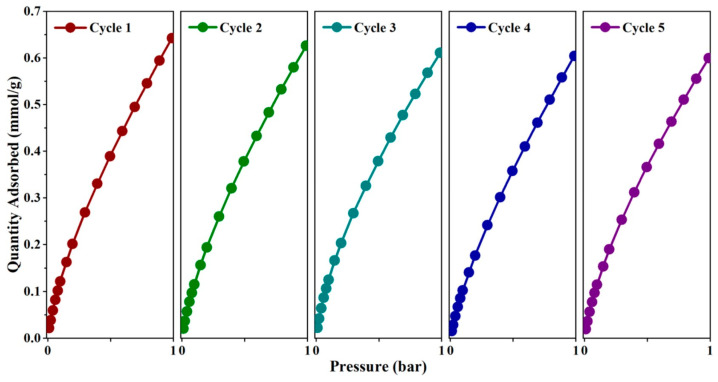
Cyclic CO_2_ adsorption isotherms of porous SiO_2_ at 298 K and 1 bar. Reprinted with permission from [217].

Many studies focus on CO_2_ adsorption by porous SiO_2_ since CO_2_ is the most important “greenhouse” gas derived from the combustion of power plants, metallurgy, cement, chemical production, and human activities, which produces negative effects, such as global warming and the relative environmental consequences [216]. For this purpose, Xu et al. [217] reported the synthesis of mesoporous SiO_2_ from the biomass of a power plant created from a mixture of wheat straw, corn straw, and forestry waste (in 5:2:3 ratio). Mesoporous SiO_2_ was synthetized by the addition of CTAB to the biomass at hydrothermal conditions (120 °C, 24 h). SiO_2_ showed a SSA of approx. 500 m^2^ g^−1^ and average pore diameter of ca. 3.5 nm; a maximum adsorption capacity of 0.7 mmol g^−1^ in optimal conditions (at 298 K, 1 bar after a 28 h hydrothermal reaction time) was observed (Figure 8) [217]. The authors explained that, while a brief hydrothermal time reduces the porosity of the mesoporous SiO_2_, the hydrothermal treatment increases caused a mesoporous SiO_2′_s entry diameters’ increase, enhancing its ability to sorb CO_2_ (Figure 9) [217].

**Figure 10:**
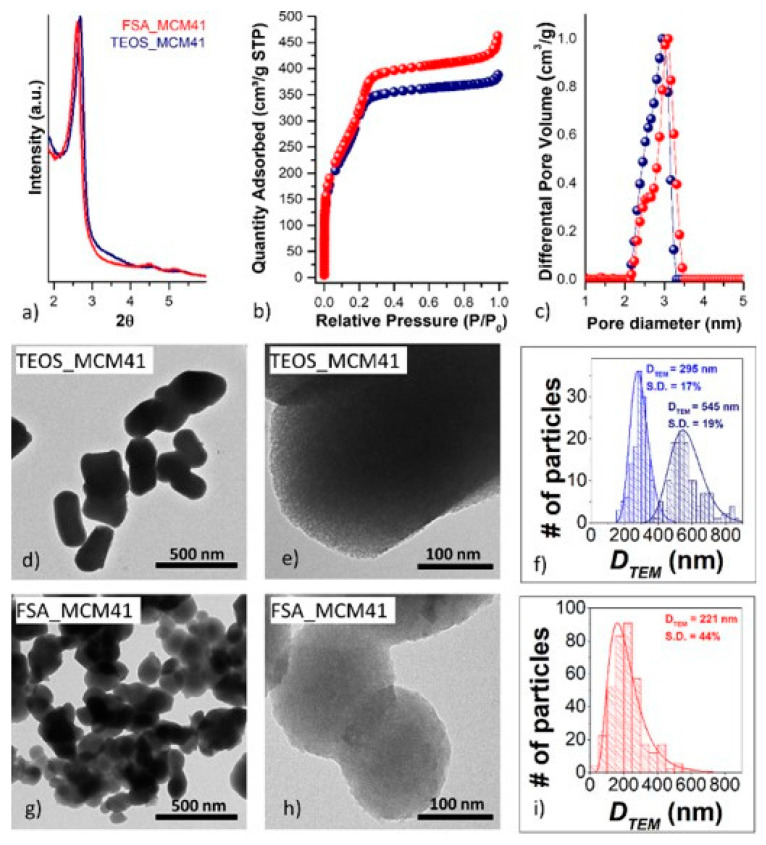
SA-XRD patterns: (**a**), N_2_-physisorption isotherms (**b**), DFT-calculated pore size distributions (**c**), TEM micrographs (**d**,**e**,**g**,**h**) and particle size distribution calculated by TEM with about 150 and 300 particles in the case of TEOS_MCM41 and FSA_MCM41 samples, respectively (**f**,**i**) of the samples TEOS_MCM41 (**d**–**f**) and FSA_MCM41 (**g**–**i**). Reprinted with permission from [64].

In the case of SiO_2_ from the secondary-product FSA, Vacca et al. [64] evaluated the possibility of using mesoporous SiO_2_ as a sorbent for removing H_2_S from syngas upon the modification of the SiO_2_ particles with iron oxide nanoscopic systems. The mesoporosity was achieved through the addition of CTAB as an organic template before the ammoniation step (i.e., molar ratios of the reactants were 6:120:1:14:4400 for FSA:ammonia:CTAB:ethyl acetate:water, respectively). CTAB removal was reached by either calcination under static air at 550 °C for 4 h, or by solvent extraction with EtOH, the latter demonstrating the possibility of recovering and reutilizing CTAB (Figure 10) [64].

SSAs for mesoporous samples were around 1100 m^2^ g^−1^; thus, comparable with those obtained using a traditional process starting from alkoxides (i.e., TEOS). The synthesis of the Fe_2_O_3_-based nanocomposite was performed via the so-called two-solvent incipient impregnation technique. An aqueous solution of iron(III) nitrate was carefully added dropwise to MCM-41 from FSA dried at 120 °C and calcined at 500 °C. Porous Fe_2_O_3_-SiO_2_ from FSA shows a sulfur retention capacity of approx. 20mg_s_ g_sorbent_^−1^, under tests of sulfidation–regeneration cycles, with performances similar to the reference material conventionally obtained using TEOS as a silica precursor [64]. Furthermore, the use of FSA as a source of silicon, the possibility of also producing CaF_2_ as a value-added product, and the recovery of both ammonia and CTAB could play a crucial role in the design of an efficient process that meets the requirements of both green chemistry and a blue economy (achieving environmental and economic sustainability).

**Figure 11:**
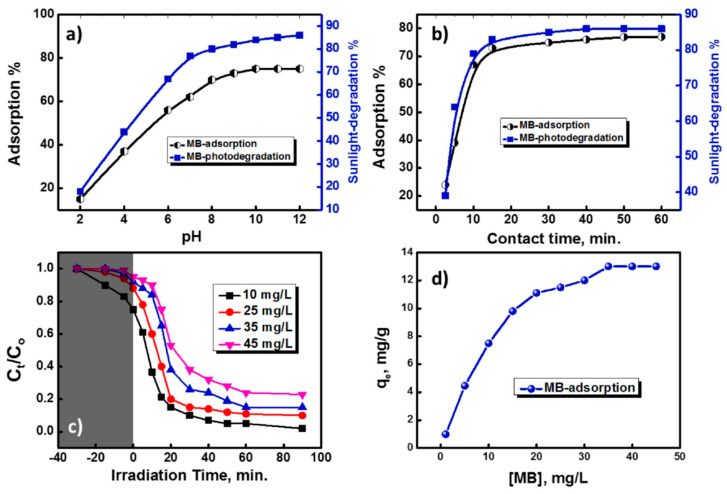
Effect of pH (**a**), contact time (**b**), sunlight-driven photo-catalytic degradation using mesoporous ZrO_2_-SiO_2_ nanocomposite (with porous SiO_2_ from rice straw) (**c**), and MB concentration on MB adsorption (**d**). Reprinted with permission from [218].

In the work of Motawea et al. [218], instead, hybrid ZrO_2_:SiO_2_ systems obtained from rice straw were tested as both an adsorbent and photo-catalyst against methylene blue (MB) dye. First, rice straws were acid leached and calcined at 700 °C to obtain SiO_2_-rich ashes, which subsequently reacted with a 1M NaOH solution at 70 °C to produce sodium silicate. The mesoporous ZrO_2_-SiO_2_ composite was prepared by mixing ZrO_2_ NPs into the silicate solution by stirring and then adding dropwise a 50% sulfuric acid solution until the formation of ZrO_2_-SiO_2_ at pH 8, which, finally, was dried and calcined at 550 °C for 5 h under an air atmosphere. The ZrO_2_-SiO_2_ nanocomposite was tested as an adsorbent/catalyst in the photo-catalytic degradation of the MB dye when exposed to sunlight, exploring the impact of different parameters, including pH, contact/irradiation times, substrate concentration, adsorbent/catalyst dosages, and temperature, demonstrating a removal capacity of approx. 75–85% of the organic dye at an alkaline pH (ca. 10) (Figure 11) [218].

**Figure 12:**
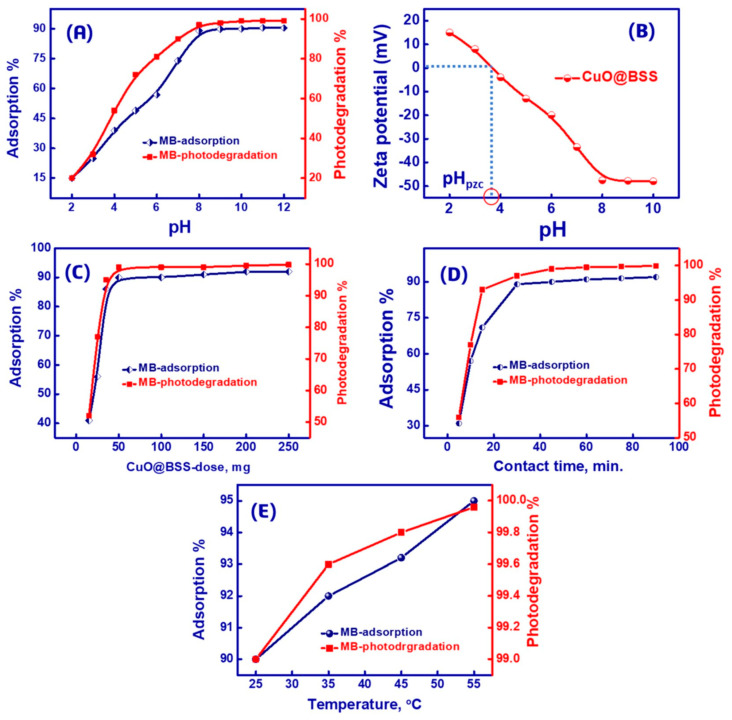
(**A**) Impact of pH on MB adsorption and photodegradation using mesoporous CuO-SiO_2_ nanocomposite (with SiO_2_ from barley straw); (**B**) zeta potential of CuO-SiO_2_ photo-catalyst/adsorbent to assess the charge of the surface at several pH values (pH 1.0–10.0), (**C**–**E**) effect of different parameters on CuO-SiO_2_ activity. Reprinted with permission from [219].

In fact, ZrO_2_ NPs efficiently produce free radicals as protons and hydroxyl radicals, where hydroxyl radicals act as efficient oxidizing agents to decompose MB molecules into smaller species, such as CO_2_, and H_2_O, under sunlight irradiation conditions. On the other hand, SiO_2_ significantly improves the system’s efficiency thanks to its large SSAs and mesoporosity, providing a rapid diffusion of the dye along the hybrid surface. In another work of the same group [219], the authors investigated the synthesis of the hybrid CuO:SiO_2_ system obtained from barley straw. The synthesis proceeded with a similar procedure and condition [218], namely, barley straws were acid leached, calcined at 700 °C and, and, subsequently, the SiO_2_-rich ash was treated with 1M NaOH at 70 °C. Then, CuO particles were added to the sodium silicate solution, maintained under stirring for 12 h, and then calcined at 550 °C for 5 h to obtain the CuO-SiO_2_ composite. Adsorption and photo-degradation trials were performed under UV irradiation at RT using a 70 W UV lamp. Figure 12 presents the effect of different parameters on the CuO-SiO_2_ removal efficiency toward MB dye as the target molecule [219].

CuO-SiO_2_ from barley straw exhibited promising and convenient adsorbent/photocatalyst properties at pH 10 with respect to dye degradation by treating contaminated water with UV irradiation.

**Figure 13:**
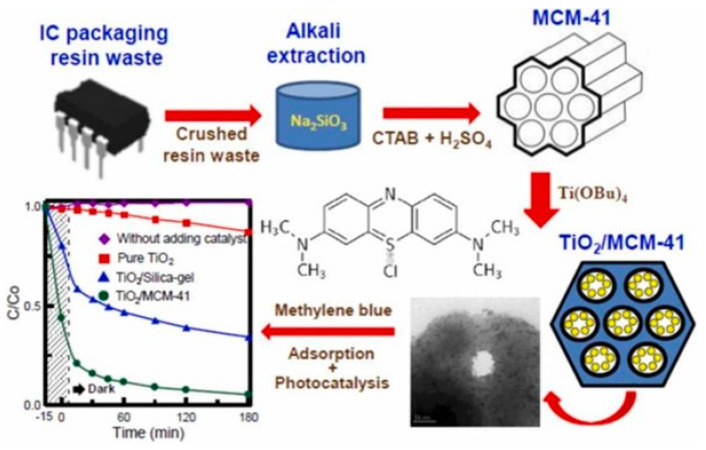
Graphical representation of the synthesis and application of SiO_2_-TiO_2_ obtained from E-waste. Reprinted with permission from [220].

Concerning the possibility of using porous SiO_2_ from waste sources as a catalyst support, Liou et al. [220] reported the synthesis of a mesoporous MCM-41 SiO_2_-TiO_2_ nanocomposite obtained from electronic waste (Figure 13). The raw material used in the study was resin waste from electronic packaging. The starting material was ground into powder and burned under an air atmosphere at 800 °C to eliminate the organic fraction. Furthermore, sodium silicate was obtained by alkaline extraction performed by using a 4M NaOH solution at 100 °C for 6 h. Mesoporous MCM-41 SiO_2_ particles were obtained following an autoclave treatment in the presence of a sodium silicate solution and CTAB heated at 100 °C for 48 h. After filtration, washing, and drying, the resultant product (with a purity of 99.8%) was calcined under an air atmosphere at 550 °C for 6 h. TiO_2_ NPs were synthesized by dissolving a fixed amount of Ti(OBu)_4_ in a mixed solution of isopropanol and acetylacetone under constant-stirring conditions. Isopropanol and deionized water were then slowly added to the titanium solution. Following the [Ti(OH)_4_] formation, the white precipitate was dissolved in deionized water in the presence of the MCM-41 SiO_2_ substrate. Thereafter, 0.5M of nitric acid was added dropwise, and the mixture was heated with reflux at 85 °C for 12 h. The solid was washed with water, centrifuged, and dried in an oven. The specimens were collected and then calcined at different temperatures between 300 and 700 °C for 2 h in an air environment.

The photocatalytic performances of the nanocomposite were monitored against MB dye as the target substrate, reaching the best efficiency in the case of the nanocomposite with an SiO_2_:TiO_2_ molar ratio of 7:3 obtained after calcination at 700 °C for 2 h. A higher catalytic activity was observed in the case of the nanocomposite with respect to bare TiO_2_, probably due to the higher SSA induced by the mesoporous SiO_2_ and the presence of anatase TiO_2_ NPs with ca. 30 nm diameter homogenously dispersed on the SiO_2_ surface.

To sum up, the overview presented in this paragraph clearly demonstrated that the studies available on the literature describing the performances and properties of SiO_2_ NPs obtained from waste and secondary products as alternative sources were comparable to their analogs obtained from traditional and commercially available routes. However, it is important to note that the majority of these studies are in the preliminary bench-scale phase, at present, and are awaiting further investigation and scaling up.

Apart from the few limitations, such as the presence of impurities overcoming the specific purification process, porous SiO_2_ particles obtained from waste sources are undoubtedly promising substrates, not only in terms of their experimental performances in many application fields, but also in view of a more sustainable production and waste valorization.

The analysis of the literature on this very appealing topic highlights that this technology (that is easily scalable) is beneficial and economically advantageous. Even though the production cost of porous SiO_2_ might be higher compared to other materials, its higher added-value compensates for this drawback. The higher added-value indicates that the end product or application of porous SiO_2_ can command a higher price in the market, potentially leading to increased profitability.

Moreover, the integration of this technology with conventional industrial chemical production, specifically FSA-derived SiO_2_, can further enhance its economic advantages. By combining this new technology with the existing chemical production processes, it is possible to create synergies, reduce overall costs, and improve the overall efficiency of the production process (e.g., FSA-derived SiO_2_).

Although it is important to underline that, in the case of mesoporous materials treated in this review, some drawbacks and limitations of their applications in large quantities were present. These limitations were primarily associated with the challenges of transitioning the synthesis process from the laboratory scale to industrial settings, which involved dealing with issues, such as pollution resulting from calcination, the potential hazards of using organic templates, and the high costs associated with these templates. Nevertheless, there are several already known studies focusing on addressing these concerns by exploring the recovery and reuse of templates [221].

## 4. Conclusions

In recent years, the re-utilization of agricultural and industrial waste/byproducts as sources for the production of high-added-value chemicals has become a challenging topic, attracting both the interest of researchers and capital goods from the industry, as it favors the conservation of natural resources and reduces the landfill phenomenon. However, even if desirable, overcoming the linear economy paradigm with a more circular and environmentally valuable one is not always a simple and direct process.

In the present study, the most recent progress on the synthesis of SiO_2_ systems (eventually mesoporous) from different waste and secondary products was critically discussed in this study. After providing a classification based on the origin of the starting waste/byproduct, the most common synthesis procedures involving these unusual precursors were extensively analyzed, highlighting the major factors influencing the morphology of the final products, the key role played by the initial precursor’s composition, and the consequent pre-treatment conditions mandatory for achieving highly pure SiO_2_. Based on the analyzed literature, the following two relevant conclusions can be assumed, namely:

(I) The majority of the case studies relies on the use of agricultural (e.g., rice husk) and industrial waste (primarily deriving from mills and mining operations).
(II) The recent trend is the valorization of secondary products as a potential source of silica. In this context, FSA is a promising candidate as it was recently recognized as being a valuable source of Si-containing commodities. Even if the production of SiO_2_ from FSA is still a challenging route, this process potentially guarantees the improvement of environmental issues related to FSA disposal, which is mostly performed by direct neutralization into the sea (with environmental consequences easy to assume).

## 5. Future Perspectives

The reutilization of agricultural/industrial waste and byproducts is an important economic/industrial strategy to achieve more sustainable industrial development goals. However, the analysis of the state-of-the-art literature clearly shows that simply starting from a sustainable source is not always directly proportional to achieving desirable sustainability. In fact, several concerns are still related to these unconventional approaches:

(I) The difficulties of reaching uniform experimental and procedural parameters. This criticality is typically associated with processes based on the re-use of agricultural wastes. Biomasses are characterized by showing an intrinsic variability in terms of the type and origin of the biomass, seasonality, and purification processes used for the recovery of the given waste, which usually contains a high content of not easily removable impurities.
(II) The second concern, instead, is cross-sectional for all the alternative processes discussed here, which are the environmental and handling issues related to the use of strong acids and bases. This last assumption is a clear example of how the valorization of sustainable resources for producing high-added-value chemicals may not be a sustainable alternative if the adopted alternative process requires the use of hazardous procedures analogous to traditional procedures. Therefore, a critical balance between the benefits and drawbacks is always mandatory.
(III) The last concern is relative to the criticalities associated with the templating procedure necessary for obtaining porous SiO_2_, which remains analogous to the traditional routes.

According to a recent study by Razak et al. [222], there are still significant knowledge gaps that should be addressed before transitioning the production of waste-derived SiO_2_ from the laboratory level to the real world. Among these gaps, the maintenance of SiO_2_ characteristics and quality consistency independently from the starting source is surely one of the major challenges. The future of this topic is fluid and subject to important changes. The analysis of the literature discussed here evidences that, even if potentially interesting and promising, the valorization of agricultural and industrial waste/byproducts for obtaining SiO_2_ remains a complex field of research that deserves an improvement for becoming industrially scalable. In particular, the simple choice of alternative, “greener”, valuable residues as feedstock for the industrial production of silica is not enough if the adopted processes are both environmentally and economically unsustainable. In this context, the involvement of relevant industries, such as Continental, Goodyear, and Pirelli, can be an important push towards an increase in industrial feasibility. However, independently from this point, the possibility of valorizing industrial byproducts remains the more reliable choice for achieving rapid industrial integration. The scientific literature reviewed here clearly showed that the future of this research field is clearly promising and a current need. The recommendation is trying to focus not simply on the different potential sources of SiO_2_, but more effectively on the importance of investigating alternative (milder)-process conditions, which is the real mandatory need, for achieving important and immediate results for industrial scalability. According to the present authors, the design of the integrated processes with zero waste and fully valorized routes will likely be a future trend, as sustainability does not necessarily require brand new processes; it also passes from the optimization of the ones available at present (as in the case of FSA valorization).

## Figures and Tables

**Table 1:** Composition of inorganic residues from different agricultural waste materials.

Agricultural Waste	SiO_2_ (wt.%)	K_2_O (wt.%)	Na_2_O (wt.%)	CaO (wt.%)	MgO (wt.%)	Al_2_O_3_ (wt.%)	Fe_2_O_3_ (wt.%)	ZnO (wt.%)	MnO_2_ (wt.%)	SO_3_ (wt.%)	Ref.
Rice husk ash	89.61	2.53	0.16	1.52	0.56	0.36	0.90	-	-	-	[87]
	79.63	3.83	7.04	2.36	-	1.86	1.04	0.09	-	-	[84]
	93.10	0.04	0.96	1.52	0.65	0.07	-	-	-	0.09	[82]
Sugar bagasse ash	75.96	5.24	-	3.85	2.53	2.24	5.08	-	-	-	[75]
Wheat straw	88.09	1.84	0.36	2.22	0.99	-	0.48	-	0.02		[77]
	10.80	2.01	-	0.28	0.18	-	0.37	-	-	-	[78]
Corncob ash	27.80	18.49	-	14.03	9.50	5.70	4.69	-	-	-	[80]
	68.70	4.56	4.60	9.50	5.20	-	3.44	-	0.14	0.08	[79]
Bamboo leaf ash	74.41	-	10.30	6.51	4.07	1.13	1.55	-	0.07	1.44	[76]

**Table 2:** Chemical composition of nanoscopic SiO_2_ obtained from barley husk. Reprinted with permission from [100].

Oxides	Composition of SiO_2_ Obtained from Barley Husk at Different Treatment Conditions			
	at 400 °C (%)	at 500 °C (%)	at 600 °C (%)	at 700 °C (%)
SiO_2_	93.0	93.5	93.5	94.1
CaO	1.0	0.9	0.9	0.7
MgO	0.9	0.8	0.8	0.8
K_2_O	2.1	2.0	2.0	1.9
Fe_2_O_3_	0.5	0.3	0.3	0.3
P_2_O_5_	0.6	0.6	0.6	0.6
Al_2_O_3_	0.7	0.7	0.7	0.7
B_2_O_3_	1.2	1.2	1.2	0.9

**Table 3:** General chemical composition of different mining wastes.

Mining Waste	SiO_2_ (wt.%)	K_2_O (wt.%)	Na_2_O (wt.%)	CaO (wt.%)	MgO (wt.%)	Al_2_O_3_ (wt.%)	Fe_2_O_3_ (wt.%)	ZnO (wt.%)	TiO_2_ (wt.%)	CuO (wt.%)	Ref.
**Copper ore tailing**	68.23	-	1.43	5.27	1.44	5.01	10.16	-	-	0.12	[133]
**Iron ore tailing**	50.88	-	.	3.26	0.59	13.62	25.18	-	0.28	-	[134]
	76.70	0.26	0.30	1.48	2.93	1.95	15.77	0.01	0.03	0.01	[135]
	82.26	-	-	0.57	-	0.80	14.37	-	0.02	-	[136]
**Gold mine tailing**	89.03	1.53	0.17	0.21	0.26	4.43	1.42	-	0.44	-	[137]
	57.70	3.74	3.02	5.97	3.77	17.20	6.33	-	0.93	-	[138]

**Table 4:** List of different types of mesoporous SiO_2_ NPs and their characteristic properties. For the acronyms, please refer to Figure 5. Reprinted with permission from [205].

Mesoporous SiO_2_ NP Family	Acronym	Pore Symmetry	Pore Size (nm)	Pore Volume (cm^3^ g^−1^)
M41S	MCM-41	2D hexagonal	1.5–8	>1.00
	MCM-48	3D cubic	2–5	>1.00
	MCM-50	Lamellar	2–5	>1.00
SBA	SBA-11	3D cubic	2.1–3.6	0.68
	SBA-12	3D hexagonal	3.1	0.83
	SBA-15	2D hexagonal	6.0	1.17
	SBA-16	Cubic	5–15	0.91
KIT	KIT-5	Cubic	9.3	0.45
COK	COK-12	Hexagonal	5.8	0.45
FDU	FDU-12	3D cubic	10–26	0.66

## Data Availability

Not applicable.

## Abbreviations

BR	butadiene rubber.
CTAB	cetyltrimetilammonium bromide.
FSA	hexafluorosilic acid.
GO	graphene oxide.
MB	methylene blue dye.
NPs	nanoparticles.
PEG	polyethylene glycol.
RT	room temperature.
SSA	specific surface area.
SBR	styrene butadiene rubber.
TEOS	tetraethyl orthosilicate.
TMSO	tetramethyl orthosilicate
